# The Application Potential and Advance of Mesenchymal Stem Cell-Derived Exosomes in Myocardial Infarction

**DOI:** 10.1155/2021/5579904

**Published:** 2021-06-01

**Authors:** Xianyun Wang, Yida Tang, Zhao Liu, Yajuan Yin, Quanhai Li, Gang Liu, Baoyong Yan

**Affiliations:** ^1^Cell Therapy Laboratory, The First Hospital of Hebei Medical University, Shijiazhuang, China; ^2^Department of Cardiology, The First Hospital of Hebei Medical University, Shijiazhuang, China; ^3^Department of Cardiology, Peking University Third Hospital, Beijing, China; ^4^Traditional Chinese Medicine Processing Technology Innovation Center of Hebei Province, College of Pharmacy, Hebei University of Chinese Medicine, Shijiazhuang, China

## Abstract

Myocardial infarction (MI) is a devastating disease with high morbidity and mortality caused by the irreversible loss of functional cardiomyocytes and heart failure (HF) due to the restricted blood supply. Mesenchymal stem cells (MSCs) have been emerging as lead candidates to treat MI and subsequent HF mainly through secreting multitudinous factors of which exosomes act as the most effective constituent to boost the repair of heart function through carrying noncoding RNAs and proteins. Given the advantages of higher stability in the circulation, lower toxicity, and controllable transplantation dosage, exosomes have been described as a wonderful and promising cell-free treatment method in cardiovascular disease. Nowadays, MSC-derived exosomes have been proposed as a promising therapeutic approach to improve cardiac function and reverse heart remodeling. However, exosomes' lack of modification cannot result in desired therapeutic effect. Hence, optimized exosomes can be developed via various engineering methods such as pharmacological compound preconditioned MSCs, genetically modified MSCs, or miRNA-loaded exosomes and peptide tagged exosomes to improve the targeting and therapeutic effects of exosomes. The biological characteristics, therapeutic potential, and optimizing strategy of exosomes will be described in our review.

## 1. Introduction

Myocardial infarction (MI) is a devastating disease with high morbidity and mortality caused by the irreversible loss of functional cardiomyocytes due to the restricted blood supply. Many attempts have been made to improve the cardiac function and reduce mortality of MI, while only the regeneration of new myocardial tissue to replace the damaged tissue was the ultimate goal for restoring cardiac function. Mesenchymal stem cells (MSCs) have attracted wide attention due to their high reproductive activity, multilineage differentiation potential to mesoderm or nonmesoderm tissues, immunomodulatory properties, and broad-spectrum releasing of cytokines which endow themselves as a promising candidate to mitigate MI and subsequent heart failure (HF) [1, 2]. Previous preclinical or clinical studies of MSC administration in MI have confirmed the cardiac improvement and regeneration effects [3]. However, the low retention and poor cell survival rates in the myocardium hamper the therapeutic effects of MSC which explain that the repair mechanisms in cardiac function and structure mainly rely on the paracrine action of MSCs other than in situ differentiation into cardiomyocytes. In recent years, many strategies have been provided to improve the functional benefit of transplanted stem cells through drug pretreatment [4, 5], genetic modification [6], ischemic preconditioning, and improvement of the infarcted microenvironment to boost cardiac regeneration and angiogenesis and inhibit fibrosis progression and anti-inflammatory action which are closely related to the communication between infarcted microenvironment and administrated stem cells. Multiple experiments have underlined the powerful effective factors released from transported mesenchymal stem cells.

The paracrine factors released from MSCs have been proved to possess the major regulation effect on myocardial regeneration and angiogenesis [7, 8] through promoting the proliferation of cardiomyocytes and endotheliocytes and restraining apoptosis of cardiomyocytes [9, 10]. Indeed, MSC-conditioned media and coculture systems have exhibited notable promotion on the proliferation of cardiomyocytes and endotheliocyte and inhibition effects of hypoxic ischemia or oxidative stress-induced cardiomyocyte apoptosis resulting in enhanced myocardium viability and angiogenic ability. The cytokine profiles released from MSCs have been detailedly developed to exercise their repair functions such as vascular endothelial growth factor (VEGF), stromal cell-derived factor 1 (SDF-1), and insulin-like growth factor 1 (IGF-1). The single delivery of VEGF and IGF-1 has shown obvious cardiac therapeutic effects in preclinical and clinical studies, while only unstable and marginal efficacy was observed which may be related to the short half-life of cytokines in vitro and unascertained effective doses.

Recently, emerging lines of evidence have pointed out the powerful therapeutic effects of MSCs in the recovery of cardiac function similar to their parent MSCs mainly through secreting extracellular vesicles (EVs) such as exosomes, microvesicle (MV), and apoptotic body to transport multitudinous molecules from donor cells to recipient target cells [11–13]. The advantageous characteristics of MSCs raise the possibility that EVs coming from the original MSCs should hold similar benefits in cardiac protection and repairment. Timmers et al. furtherly confirmed that only factors more than 1000 kDa could repair the ischemia-reperfusion injury in a mouse model of MI and verified that these factors are exosomes released from MSCs [14]. Exosomes with lipids, proteins, mRNAs, and microRNAs packaged in have been extensively demonstrated to modulate gene expression and influence the phenotype of recipient cells in intercellular communication, signal transduction, and immune regulation [15, 16]. Additionally, exosomes also display unique advantages in boosting the repairing of heart function, such as lower malignant potential, more potent immunomodulatory effects, easier storage and transplantation, weaker reject reaction, and no involvement in cell apoptosis compared to stem cell transplantation [17]. What is more, exosomes are less likely to encounter the problem of pulmonary first-pass effect [18]. However, due to unoptimized MSC-Exos limiting the final efficacy in MI therapy, several tactics have been raised as a cell-free strategy to improve the retention or cardiac preservation effects for better therapeutic effects [19]. Nowadays, exosomes are emerging as a new potential selection for postinfarction repairment. This review is aimed at discussing the current knowledge on biological characteristics and the mechanisms of exosomes, the problems, and the optimization strategies of exosomes to improve therapeutic effect and disclose their huge therapeutic potential for MI treatment.

## 2. Biological Characteristics of Mesenchymal Stem Cell-Derived Exosomes

Extracellular vesicles (EVs) are bilayer lipid membrane-cloaked subcellular vesicles released by almost all cell types such as platelets, lymphocytes, stem cells, glial cells, muscle, and adipocyte cancer cells and also present in all body fluids including blood, urine, milk, and spit [20–24]. It has been verified that EVs participate in a range of processes through transporting the contents to targeted cells including regulating the homeostasis and promoting host-defense reaction in physiology [21, 25, 26] and supporting cancer development and progression in pathology [27]. There are mainly three subtypes of EVs known as exosomes, microvesicles, and apoptotic bodies which are usually differentiated by the biogenesis mechanism and their sizes [21, 28].

Exosomes are small extracellular vesicles containing important bioactive constituents with a diameter ranging from approximately about 40 to 160 nm [29, 30]. They are formed by the release of multivesicular bodies which are derived from the inward budding of the plasma membrane [31]. Thus, MSC-Exos express not only the ubiquitous surface marker of exosomes, such as tetraspanins (CD9, CD63, and CD81), Alix, and Tsg101, but also the MSC membrane protein of some adhesion molecules including CD29, CD44, and CD73 [32, 33]. Various isolation methods of exosomes are usually based on the basic biological properties including size, density, and membrane-bound antigens like ultracentrifugation, ultrafiltration, size exclusion chromatography, precipitation, immunoaffinity-based capture, and microfluidic technologies [34–37].

Under the internalization of the extracellular membrane, multiple biologically active molecules including proteins, lipids, RNAs, microRNAs, long noncoding RNAs (lncRNAs), transfer RNA (tRNA), genomic DNA, cDNA, and mitochondrial DNA (mtDNA) are packaged in exosomes that are closely related to the cell type, stimulus condition, disease status, and preconditioning or genetic manipulation of the parent MSCs [38–43]. Exosomes can easily pass the contents of parent cells across the cell membrane by endocytic uptake, direct cell membrane fusion of exosomes to receipt cells, or the interaction of ligand and receptor [44] and transport biologically active macromolecules to target cells [45]. These communications indicated the pivotal role of exosomes regulating immune effect, angiogenesis, or tissue repair in physiologic or pathologic conditions. The content of the exosomes derived from different cells and organisms has been broadly described in the ExoCarta database [32]. It has been proved that MSC-derived exosomes keep a remarkable and similar immunoregulatory property to their parent cells [46], and this was further confirmed by a clinical trial of MSC-Exos administration in patients with intestinal graft-versus-host disease (GVHD) grade IV resulting in a significant amelioration of symptoms [47]. The similar cardioprotection effect of MSCs and their derived exosomes (MSC-Exos) enable MSC-Exos to be an important and potential therapeutic way to treat diseases.

## 3. The Advantages and Disadvantages of MSC-Exos Treatment in MI

Growing evidence has confirmed several advantages of exosome treatments compared to cell-based treatments. The key issues limit the progression of MSC administration in MI due to the low retention and survival rates in the infarcted microenvironment. Exosomes as the most crucial paracrine production of MSCs could act as a cargo to transport the bioactive components to the surrounding cells and circulatory system and even pass across the blood-brain barrier [48, 49]. This nanoparticle could also endow their potential to surmount the primary capillary bed after intravenous transplantation allowing injected exosomes to reach the damaged myocardium.

Compared to the preclinical studies, although there are only a few clinical trials of exosome administration in heart allograft recipients [50], cancer patients [51, 52] have demonstrated positive effects of MSC-Exos, and no side effects were observed implying the safety, tolerability, and efficacy of exosome treatment in principle [53–55]. MSC transplantation may lead to more safety concerns than exosome treatment with respect to tumorigenicity and immunological rejection [56]. A safety evaluation study of human umbilical cord mesenchymal stromal cell-derived exosomes intravenously given to rabbits, guinea pigs, and rats showed no adverse effects not only on the liver and renal function but also on hemolysis, vascular and muscle stimulation, systemic anaphylaxis, pyrogen, and hematology indexes indicating applicability and toleration of exosome released from human umbilical cord mesenchymal stromal cells in future clinical therapy [57]. Exosomes can be locally transplanted with controlled and defined dosage in space and time, whereas implantation of living cells may suffer from apoptosis or death and not fully home to the damaged site [58, 59]. Additionally, exosomes can directly transport modified bioactive molecules provided by manipulated or preconditioned source cells to target cells [60]. Moreover, exosomes as nonviable vesicles have been proved to lower the risk of long-term side effects, such as thrombogenesis, arrhythmia, calcification, multidirectional differentiation, and tumor [61, 62]. Furthermore, exosomes are more stable, are easier stored and transported in vitro, and can be kept for approximately 6 months at -20°C without change in their biochemical activities [33]. However, several disadvantages including laborious and inefficient isolation methods, unrenewable capacity, and short-term use only also limit the clinical application of exosomes [63, 64]. It has also been reported that intravenously transplanted exosomes missed out on the targeting location and preferentially accumulated in the liver which was mainly due to abundant resident macrophages. Blocking the phagocytosis of exosomes by macrophages could effectively increase the exosomes targeting location toward the heart [65]. Therefore, apart from the obvious disadvantage of the isolation process, stem cell-derived exosomes have displayed a powerful advantage on the therapeutic effect, which enabled improved exosome isolation method to come out [34].

## 4. Therapeutic Potential of MSC-Exos in MI

Exosomes have shown remarkable inhibition effect on cell apoptosis, fibrosis and inflammation, and promotion of angiogenesis for myocardial repair. MicroRNAs (miRs) loaded in exosomes have been considered the major components for these beneficial functions indicating a potential alternative tool for cell-free therapy for cardiovascular diseases [30, 66, 67]. Thus, some major noncoding RNAs and the functional component will be reviewed in the four aspects of cardiac tissue preservation, enhanced angiogenesis, limited inflammation, and ECM remodeling (seen in Table 1).

### 4.1. Myocardial Preservation

Increasing present studies have shown the cardioprotective roles of miRNAs secreted by transplanted mesenchymal stem cells from various sources such as bone marrow, adipose tissue, umbilical cord, umbilical cord blood, and endometrium. MSC-Exos have been reported to promote proliferation and inhibit apoptosis of H9C2 cells induced by H_2_O_2_ and prevent TGF-*β*-induced transformation of fibroblast to myofibroblast [17]. The *in vivo* experiments proved that MSC-Exos (20 *μ*g/20 *μ*L) and MSCs (1∗10^6^ cells) could markedly increase LVEF (MSC-Exo group: 59.66 ± 4.38%, MSC group: 50.23 ± 3.45%, and control: 30.77 ± 4.13%. *P* < 0.05), reduce fibrosis (*P* < 0.05), and reduce inflammation (*P* < 0.05) than the control group (20 *μ*L PBS) in a rat model of MI. Tail vein injection of MSC-Exos into I/R mice 5 minutes prior to reperfusion significantly reduced infarct size and both local and systemic inflammation one day after I/R injury compared to saline treatment by directly targeting cardiac cells. MSC-Exo could effectively prevent LV dilation and improve cardiac function at 28 days after myocardial I/R injury associating with the increased level of ATP and NADH and phosphorylated-Akt and phosphorylated-GSK-3*β* and decrease in phosphorylated-c-JNK in ischemic/reperfused hearts after MSC-Exo injection [68]. BMSC-Exos (80 *μ*g/200 *μ*L) were injected at four different sites in the myocardium bordering the infarcted zone of MI rats resulted in galvanizing impact in tube formation and inhibitory effect on T lymphocyte proliferation in vitro experiment. In vivo animal experiments further confirmed the positive impact of BMSC-Exos on increased LVEF, reduced infarct size, preserved cardiac systolic and diastolic performance which finally enhanced the density of new functional capillary, restrained inflammation response, and improved heart function after ischemic injury [61].

Recently, some pivotal miRNAs have been reported to promote cardiac preservation through regulating and benefitting the myocardial cells from suppressing cell apoptosis and oxidative stress and promoting cardiac repair and regeneration. Exosomes secreted by BMSCs like miR-125b (mediating p53-Bnip3 signaling) [69] and miR-25-3p (directly targeting the proapoptotic genes FASL and PTEN) [70] have been, respectively, proved to confer cardioprotective effects and suppress inflammation in the myocardial repair process. Exosomes from AD-MSCs can deliver miR-146a (through interacting with the 3′-untranslated region of EGR1) [71] and miR-221/222 (via the PUMA/ETS-1 pathway) [72] exerting significant antiapoptosis of cardiomyocytes and suppressing inflammatory response and fibrosis. miR-21 in EnMSCs showed superior cardioprotection through antiapoptotic and angiogenic effects by enhancing cell survival through the miR-21/PTEN/Akt pathway [73]. Additionally, exosomes from miRNA-overexpressing MSCs like miR-126 [74], miR-210 (by targeting AIFM3) [75], and miR-133 (targeting snail 1) [76] or miRNA-transfected exosome miR-338 (through regulating MAP 3K2/JNK signaling) [77] have also revealed to inhibit cardiomyocyte apoptosis, reduce infarct size, and improve cardiac function. Exosomes obtained from GATA-4-overexpressing MSCs [43], ischemic preconditioned BMSCs [78], and hypoxia-conditioned BMSCs [79] can, respectively, enrich miR-19a (targeting PTEN to activate the Akt and ERK signaling pathways), miR-22 (by targeting methyl CpG binding protein 2), and miR-125b (through suppressing the expression of the proapoptotic genes p53 and BAK1) to ameliorate cardiomyocyte apoptosis, reduce cardiac fibrosis, and facilitate cardiac repair. Besides, lncRNA KLF3-AS1 in exosomes secreted from human MSCs can inhibit H9C2 pyroptosis and attenuate MI progression through the lncRNA KLF3-AS1/miR-138-5p/Sirt1 pathway [80]. Circular RNA 0001273 hUC-MSC-derived exosomes can remarkably inhibit the H9C2 apoptosis and promote MI repair [81]. From the reports above, we concluded that MSC-produced exosomes can shuttle many functional miRNAs, lncRNA, and circular RNA to target cells to suppress cardiomyocyte injury and exert cardioprotection roles.

### 4.2. Enhanced Angiogenesis

In addition to the affection of exosomes on cardiac myocyte function optimization, enhanced angiogenesis is another important repair mechanism contributing to improved cardiac function. miR-132 in the exosomes of BMSCs can both increase tube formation of HUVEC by targeting RASA1 and enhance the neovascularization in the peri-infarct zone and preserve heart functions in MI mice [82]. miR-210 in BMSC-secreted exosomes can also significantly improve angiogenesis by increasing the proliferation, migration, and tube formation capacity of HUVECs and contributed to cardiac function improvement post-MI [83]. miR-21 in EnMSC-derived exosomes showed superior cardioprotection through angiogenic effects via the PTEN/Akt pathway [83]. Additionally, the exosomes from *SIRT1*-overexpressing ADSCs can help to restore the function of cell migration and tube formation and recruitment of EPCs to the repair area through the Nrf2/CXCL12/CXCR7 pathway [84]. Macrophage migration inhibitory factor engineered hUC-MSC-produced exosomes also significantly enhanced proliferation, migration, and angiogenesis by delivering miR-133a-3p to HUVEC [85]. TIMP2-modified hUC-MSC-derived exosomes can promote the secretion level of Sfrp2 which contributed to HUVEC proliferation, migration, and tube formation in vitro and angiogenesis in a rat MI model [86]. Long noncoding RNA H19 packaged in exosomes from atorvastatin-pretreated BMSC-Exos have been proved to regulate miR-675 expression to activate proangiogenic factor VEGF and intercellular adhesion molecule-1 which finally exerted markable cardioprotective roles by promoting endothelial cell proliferation in AMI rat models [87]. Besides, PDGF-D in the exosomes derived from *Akt*-modified hUC-MSCs resulted in more effective improvement in MI therapy through promoting angiogenesis [88]. Therefore, MSC-derived exosomes both show powerful repair potential in improving cardiac function. Engineered MSCs are an important research focus to improve therapeutic effectiveness via promoting angiogenesis.

### 4.3. Limited Inflammation

It is well known that inflammatory reaction triggered by ischemia/reperfusion is closely related to myocardium injury and scar information. The powerful immunomodulatory effect of MSC-derived exosomes has also been concerned with the repair of ischemic cardiomyopathy. Cytokines are responsible for immunomodulation such as tumor growth factor-*β* (TGF-*β*) in the activation of regulatory T cells, interleukin 1 receptor antagonist (IL-1Ra) in the roles of M2 macrophage polarization and inhibition of B cell differentiation, prostaglandin E2 (PEG2) inducing the production of anti-inflammatory IL-10 of macrophage, and galectin-1 (Gal-1) in the inhibition role of CD4+ and CD8+ T cell proliferation [89]. miR-182 from BMSC-derived exosomes mediate Raw264.7 polarization by targeting toll-like receptor 4 mediation [90]. Overexpression of miRNA-181a in hUCB-MSC-derived exosomes suppressed inflammatory response through significantly downregulating the proinflammatory cytokines TNF-*α* and IL-6 and increasing the expression of the anti-inflammatory cytokine IL-10 in the PBMCs, promoting Treg cell polarization through targeting c-Fos and exerting a stronger therapeutic effect on myocardium I/R injury [91]. Exosomes obtained from LPS preconditioning BMSCs can strongly increase M2 macrophage polarization and attenuated the postinfarction inflammation in a mouse MI model through inhibition of the LPS-dependent NF-*κ*B signaling pathway and activation of the AKT1/AKT2 signaling pathway [92]. Melatonin-stimulated exosomes derived from AD-MSCs can promote M2 macrophage differentiation and exerted superior anti-inflammatory modulation through miRNAs miR-34a, miR-124, and miR-135b [93]. Besides, several *in vitro* studies have also confirmed the immunosuppression and anti-inflammatory action of MSC-derived exosomes like miR-10a (facilitating Th17 and Treg responses) [94] and IDO (promoting the transformation of mononuclear cells to Tregs) [95].

Therefore, MSC-secreted exosomes exhibit strong immunoregulation effects on cardiac repair and will lay an important basis for the modification and optimization of engineered MSCs for better cardioprotective effects.

### 4.4. Inhibition of ECM Remodeling

In addition to the above repair mechanism of MSC-derived exosomes, inhibition of cardiac ECM remodeling contributes greatly to the improvement of cardiac function. It has been reported that hUC-MSC-derived exosomes with miR-21, miR-23a, miR-125b, and miR-145 can suppress myofibroblast formation by inhibiting excess *α*-smooth muscle actin and collagen deposition associated with the activity of the transforming growth factor-*β*/SMAD2 signaling pathway *in vitro* and be essential for the myofibroblast-suppressing and antiscarring functions *in vivo* [102]. BMSC-secreted exosomes can enhance cardiac repair by transferring miR-29 and miR-24 to fibroblasts and inhibiting fibrosis induced by TGF-*β* [17]. Besides, TIMP2-modified hUC-MSC-derived exosomes can decrease TGF-*β*-induced MMP2, MMP9, and *α*-SMA secretion in cardiac fibroblasts and inhibit ECM remodeling [86].

Therefore, MSC-derived exosomes can serve as an important therapeutic method to repair injured tissue in ischemic heart disease through promoting cardiomyocyte survival, inhibiting fibrosis, enhancing angiogenesis, and improving the immune microenvironment. This will endow a bright therapeutic potential of MSC-Exos in various diseases in the future [103].

## 5. Engineering of Exosomes

**Figure 1:**
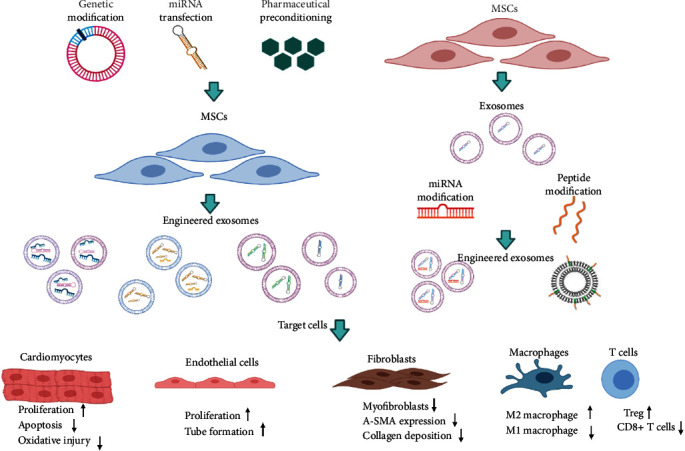
A variety of engineered modification methods of exosomes and their restorative function on different recipient cells were displayed in this figure.

The biology molecules encapsulated in exosomes are varied depending on the microenvironment surrounding them and MSCs' status. Given the advantages of higher stability in the circulation, lower toxicity, greater efficiency to transport cargoes to target cells, and easier controlled transplantation dosage, exosome treatment has been described as a wonderful and promising method in cardiovascular disease. Although exosomes could be concentrated and delivered intravenously to overcome large dosage transplantation of MSCs, the majority of injected exosomes missed out on the destination and are absorbed within the liver [65, 104, 105]. One study has tried to use approximately ten times the dose of intramyocardial transplantation to offset the nonspecific delivered exosomes [106, 107]. The low targeting to injured tissue and poor functional repairment like proangiogenesis and cardiac regeneration are the two most important factors restricting factors in improving MI. Many attempts have been made to generate engineered MSC-Exos with greater cardiovascular benefits either through genetically modified stem cells to release functional exosomes or directly decorated exosomes with homing peptides to motivate its targeted migration ability. Therefore, based on the paracrine mechanism in cardiac repairment in MI, much more efficient strategies have been broadly launched by modifying and enriching exosomal content to exert specific functions. At present, several optimization methods including preconditioning with pharmacological compounds, genetic modification, or peptide modification approaches have been broadly reported in improving cardiac function and remodeling. Thus, the influences of these factors on exosomes released by MSCs will be reviewed in the following sections (seen in Figure 1).

### 5.1. Exosomes Generated from Genetically Modified MSCs

Intramyocardial injection of GATA-4-overexpressing MSC-generated exosomes possessed powerful roles in cardiac protection or regeneration in injury myocardium through activating the miR-19a-PTEN-Akt/ERK signaling pathways [43, 108]. Another posted outcome indicated that *Akt*-modified human umbilical cord MSC- (hUC-MSC-) derived exosomes resulted in higher expression of platelet-derived growth factor D in MSC-Exos which contributed to accelerating endothelial cell proliferation, migration, and tube formation in vitro and angiogenesis in vivo [88]. A present report revealed that exosomes produced by tissue matrix metalloproteinase inhibitor 2- (TIMP-2-) modified hUC-MSCs injected in rat models via vein exhibited enhanced heart protection by decreasing cardiomyocyte apoptosis, increasing angiogenesis, and restricting extracellular matrix (ECM) remodeling partly by activating the Akt/Sfrp2 pathway [109]. Exosomes secreted by MSCs transduced with lentiviral CXCR4 significantly increased IGF-1*α* and pAkt levels and restrained the active caspase 3 level in cardiomyocytes and promoted vessel formation via enhanced VEGF expression which thoroughly contributed to neovascularization and reduced infarct size to improve cardiac remodeling [110]. Besides, the plasmid transduction of macrophage migration inhibitory factor (MIF) in bone marrow-MSCs (BM-MSCs) can generate optimized exosomes to result in decreased mitochondrial injury, reactive oxygen species generation, and cell apoptosis of cardiomyocyte under the situation of hypoxia/serum deprivation which greatly restored heart function and reduced heart remodeling in a rat model of MI [111]. These reports elaborate that genetic modification can effectively improve the function exertion of MSC-derived exosomes in various diseases. Therefore, these preclinical researches set an important foundation for the clinical application and transduction of optimized exosomes from genetically manipulated MSCs.

### 5.2. Exosomes Generated from MicroRNA Genetically Modified MSCs

miR-126-enriched exosomes derived from miR-126-overexpressing adipose-derived stem cells (ADSCs) prevented trauma by reprogramming the myocardial infarcted microenvironment, thus decreasing cardiac fibrosis and inflammation and apoptosis and increasing angiogenesis in the infarction area of AMI rats [74]. Intramyocardially injected miRNA-181a-enriched exosomes from MSCs infected with miRNA-181a-overexpressing lentiviruses in a mouse model of myocardial ischemic reperfusion injury (I/R) displayed a stronger therapeutic effect by stimulating greater immune-suppressing effect and targeting capability [91]. Direct injection of microRNA-133 overexpression MSCs around the infarcted zone displayed better cardiac function, lower inflammatory level, and less infarct size through repressing snail 1 repression [76]. miR-146a-modified adipose-derived stem cells were also confirmed to attenuate myocardial infarction-induced injury by suppressing cardiomyocyte apoptosis, inflammatory response, and fibrosis via downregulating early growth response factor 1 (EGR1) [71]. There comes one experiment to induce the cardiomyocytes to reenter into the proliferative state which is the key to restore cardiac function. Hsa-miR-590-3p secreted from BMSCs has been proposed to promote cardiomyocyte proliferation through downregulating Homer1 and Hopx expression which could inhibit cardiomyocyte proliferation [112]. Furthermore, based on the high concentration of cTnI along the infarcted region, cTnI-targeted short peptides have been designed and transfected to modify the surface of BMSCs to obtain cTnI-targeted exosomes. So, BMSC exosomes decorated with cTnI-targeted exosomes (STSMLKA) could carry Hsa-miR-590-3p to the infarcted area to promote cardiomyocyte proliferation around the peri-infarct area and finally restore heart action [108].

### 5.3. Exosomes Derived from Pharmaceutical Improved MSCs

MSCs pretreated with pharmacological agents such as atorvastatin [113, 114], rosuvastatin [115], oxytocin [116], curcumin [117], and haemin [118] have been widely performed to enhance the survival of administrated MSCs and local cardiomyocytes and stimulate angiogenesis of endothelial cells which could finally lead to further improvement of cardiac function and fibrosis. However, most pharmacological agent preconditioning may be recognized as an alternative approach to treat specific diseases either due to the limited beneficial effects on functional recovery of MI or the dynamic paracrine factors affected by surrounding culture conditions or microenvironment of injured tissue. Presently, exosomes released by ATV-pretreated MSCs have obvious therapeutic efficacy in the MI model of Sprague-Dawley rats possibly through enhancing endothelial cell function in angiogenesis. lncRNA H19 partially participated in angiogenesis to mediate cardiac repairment [87]. Thus, exosomes derived from drug prestimulation are quite different depending on the culture medium surrounding them which need to be overcome before application.

### 5.4. MicroRNAs Directly Loaded Exosomes

miRNAs, shuttled by exosomes, are among the most important molecular factors controlling cardiac repair [119–121]. Cardiac miRNAs (miR-1, miR133a, miR-208a/b, and miR-499) that are extremely related to cardiogenesis, heart function, and pathology have been abundantly detected in the myocardium. These cardiac-specific miRNAs can greatly contribute to enhancing the regenerative properties and survival action of stem cells [122]. It has also been suggested that miR-21-5p dysregulation in exosomes attained from heart failure patients weakened the regenerative activities, while restoring miR-21-5p expression helped to accelerate heart repair by enhancing functional vessels and survival of cardiomyocytes and endothelial cells via the phosphatase and tensin homolog/Akt pathway [123]. A recent report has summarised stem cell-exosomal miRNAs on cardioprotective effects either on enhancing cardiomyocyte survival and function and attenuating cardiac fibrosis (miR-19a, mirR-21, miR-21-5p, miR-21-a5p, miR-22 miR-24, miR-26a, miR-29, miR-125b-5p, miR-126, miR-201, miR-210, and miR-294) or on inducing angiogenesis (miR-126, miR-210, miR-21, miR-23a-3p, and miR-130a-3p) in ischemic myocardium after MI [66, 124, 125]. Thus, cardiac-specific miRNAs are the most promising candidates in the construction of special power exosomes which will contribute to the gene therapy in cardiovascular diseases. Therefore, built on the paracrine action of MSCs, microRNAs in exosomes secreted by MSCs have been focused on benefit of cardiac protection. That means exosomes infected with specific function miRNA mimics or antagonists may exert a better effect on cardiac function and prognosis conditions. Electroporation has been proposed as one of the most important ways to introduce therapeutic miRNA mimics or antagonists into exosomes. For example, microRNA-132 (miR-132) directly targeting p120RasGap (RASA1) transfected into BMSC-Exos using the electroporation method confirmed a higher angiogenic ability of human umbilical vein endothelial cell (HUVEC) and significantly increased vessel density and LVEF compared with the saline-treated group and normal exosome groups in the mouse MI model [82]. Besides, sonication, freeze and thaw cycles, incubation with membrane permeabilizers, and antibodies against exosomal proteins are applied in cargo loading to promote drug load [126].

### 5.5. Targeting Peptides Tagged Exosomes

With the discovery of many homing peptides targeting diseased tissues or organs, ample opportunity is coming to explore the potential of targeted gene therapy using tagged exosomes. Homing peptides have been recently put forward to decorate exosomes to further promote the targeting action toward damaged tissue parts. Cardiac homing peptides (CSTSMLKAC) have been proposed to be designed and conjugated to exosomes to create infarct-targeting MSC-Exos which led to reduced fibrosis and scar size and increased blood vessel intensity [127]. Therefore, homing peptide modification could powerfully assist precision medicine treatment of ischemic diseases.

## 6. The Prospect of MSC-Exos in Cardiovascular Disease Therapy

Exosomes from various types of cells and tissues have been strongly demonstrated and widely acknowledged their powerful effects by regulating intercellular communication. MSC-derived exosomes have growingly proved their cardiac repair effects through stimulating cardiomyocyte proliferation, vascular angiogenesis, immunoregulation, and inhibiting the progression of scar formation. However, exosomes without further modification or optimization may be the major obstacle to limit their ultimate function in MI treatment. Nowadays, many strategies have been proposed to improve their efficacy in MI therapy including gene manipulation or peptide modification of exosomes, or medicine pretreatment, or low hypoxic stimulation to increase the homing function and cardiac preservation. Although most improvements cannot be applied in clinical trials here and now, the unique clinical benefits and advantages of smaller size, easier storage, and more stability enable their promising prospect in the clinical transformation and application.

However, most investigations demonstrate the benefits of MSC-derived exosomes on heart repair without potential risks from the transportation of live MSCs. Several outcomes concluded by a few studies must arouse much attention. Firstly, all types of exosomes containing MSC-released exosomes in the bloodstream can be recognized, attacked, and engulfed by immune cells like macrophages which resulted in substantial loss of MSC exosomes [128]. Secondly, although several studies have provided clear evidence for uptake of MSC-Exos into immature myocardial cells and cell lines [129], whether MSC-Exos can carry their content to mature cardiomyocytes is still under determination now. And even more, limited uptake into cardiomyocytes in vivo also contributes to the controversy of exosome treatment in MI.

Besides, whether it is the right time to apply or push the clinical trials in MI has been commented by a present review which concluded that the clinical test should not be started and motivated before several problems are settled down including the mechanism of action, stable and easier isolation method, and high standard isolation protocols of exosomes [130–132].

Therefore, there must be a series of issues to be resolved before exosome administration in clinical trials. But I believe that exosomes secreted by MSCs must be applied in the treatment of cardiovascular diseases in the near future.

## Figures and Tables

**Table 1:** List of components of the MSC-derived exosome molecular cargo to regulate cardiac repairment published in the recent 5 years.

Diseases	Component	Type of MSCs	Target cell	Function	Reference
Cardiac preservation					
Mouse MI	miR-214	ADRC	CM	ADRC-derived exosomes inhibited cardiomyocyte cell damage under hypoxia in vitro, decreased infarcted size, and improved cardiac function through miR-214-regulated clathrin endocytosis.	[96]
Mouse MI	miR-125b-5p	HcBMSCs	CM	Exosomes from hypoxia-conditioned BMSCs can facilitate cardiac repair and ameliorate CM apoptosis through suppressing the expression of the proapoptotic genes p53 and BAK1.	[79]
Mouse MI	miR-125b	BMSCs	NMCM	MSC-derived exosomes protect NMCM from hypoxia and serum deprivation-induced autophagic flux, decreased infarct size, and improved cardiac function via miR-125b-mediated p53-Bnip3 signaling.	[69]
Mouse MI	miR-22	BMSCs	NRCMs	Exosomes from ischemic preconditioned BMSCs resulted in antiapoptotic effect on CMs due to ischemia by targeting Mecp2 and displayed reduced cardiac fibrosis.	[78]
Mouse I/R	miR-25-3p	BMSCs	CM	BMSC-derived exosomes protected CMs against oxygen-glucose deprivation-induced apoptosis by directly targeting the proapoptotic genes (FASL and PTEN) and EZH2 to confer cardioprotective effects and suppress inflammation post-I/R injury.	[70]
Mouse I/R	miR-221/miR-222	ADSCs	H9C2	ADSC-derived exosomes protect H9C2 from H_2_O_2_-induced injury *and* repair cardiac I/R injury via the miR-221/miR-222/PUMA/ETS-1 pathway.	[97]
Mouse I/R	miR-221/222	ADSCs	H9C2	ADSC-CM attenuates cardiac apoptosis and fibrosis I/R-induced cardiac injury via the microRNA-221/222/PUMA/ETS-1 pathway.	[72]
Rat MI	miR-19a	hUC-MSCs	H9C2	Exosomes secreted by hUC-MSCs protected H9C2 by miR-19a/SOX6-mediated AKT activation and JNK3/caspase-3 inhibition.	[98]
Rat MI	miR-126	ADSCs	H9C2	miR-126-enhanced ADSC-exosomes prevented myocardial damage by inhibiting apoptosis, inflammation, and fibrosis and increasing angiogenesis.	[74]
Rat MI	miR-146a	ADSCs	H9C2	miR-146a containing exosomes had more effect than the normal exosome treatment group on the suppression of AMI-induced apoptosis, inflammatory response, and fibrosis in an AMI rat model through interacting with the 3′-untranslated region of EGR1.	[71]
Rat MI	miR-210	BMSCs	NRCM	miR-210-overexpressing MSC exosomes exerted myocyte protection by targeting AIFM3 to inhibit NRCM apoptosis and reduce infarct size and improve heart function in the rat MI model.	[75]
Rat MI	miR-19a	BMSCs	NRCM	GATA-4-overexpressing MSC-derived exosomes contributed to increased CM survival, reduced CM apoptosis, and preserved mitochondrial membrane potential in CM under a hypoxic environment by targeting PTEN to activate the Akt and ERK signaling.	[43]
Rat MI	miR-338	BMSCs	H9C2	Exosomes secreted from BMSCs transfected with miR-338 mimic decreased the apoptosis of H9C2 and improved cardiac function by regulating the MAP3K2/JNK signaling pathway.	[77]
Rat MI	miR-133	BMSCs	NRCM	miR-133-overexpressing BMSC-derived exosomes inhibited hypoxia-induced NRCM apoptosis and repressed inflammatory level and the infarct size by targeting snail 1.	[76]
Rat MI	miR-29 and miR-24	BMSCs	H9C2	BMSC-derived exosomes enriched with miR-29 and miR-24 enhanced cardiac repair by promoting CM proliferation, reducing apoptosis induced by H_2_O_2_, and inhibiting fibrosis of fibroblast cell induced by TGF-*β*.	[17]
Rat MI	miR-21	EnMSCs	NRCM	EnMSCs showed superior cardioprotective effects through antiapoptotic and angiogenic effects by enhancing cell survival through the miR-21/PTEN/Akt pathway.	[73]
Rat MI	Circular RNA 0001273	hUC-MSCs	H9C2	Circular RNA 0001273 in exosomes of hUC-MSCs inhibited H9C2 apoptosis and promote MI repair.	[81]
Rat MI	lncRNA KLF3-AS1	MSCs	H9C2	Exosomes secreted from human MSCs inhibited H9C2 pyroptosis and attenuated MI progression through the lncRNA KLF3-AS1/miR-138-5p/Sirt1 pathway.	[80]
Rat MI	Sfrp2	hUC-MSCs	H9C2	TIMP2-modified hUC-MSC-derived exosomes can inhibit H_2_O_2_-induced H9C2 apoptosis and alleviate MI-induced oxidative stress.	[86]
Vitro model	miR-144	BMSCs	H9C2	BMSC-derived exosomes ameliorated CM apoptosis in hypoxic conditions by delivering miR-144 to recipient cells by targeting the PTEN/AKT pathway.	[99]
Vitro model	miR-486-5p	BMSCs	H9C2	miR-486-5p carried by BMSC-derived exosomes promoted the H9C2 proliferation and rescued H9C2 cells from hypoxia/reoxygenation-induced apoptosis by suppressing PTEN expression and activating the PI3K/AKT signaling pathway.	[100]
Vitro model	lncRNA-NEAT1	hAD-MSCs	hiPSC-derived CM	Exosomes obtained from MIF-pretreated hAD-MSCs exhibited a protective effect on CM cells from hiPSC differentiation through the lncRNA-NEAT1/miR-142-3p/FOXO1 pathway.	[101]
Enhanced angiogenesis					
Mouse MI	miR-132	BMSCs	HUVEC	BMSC-derived exosomes can both increase tube formation of HUVEC by targeting RASA1 and enhance the neovascularization in the peri-infarct zone.	[82]
Mouse MI	miR-210	BMSCs	HUVEC	miR-210 in BMSC-secreted exosomes improved angiogenesis by increasing the proliferation, migration, and tube formation capacity of HUVECs and contributed to cardiac protection.	[83]
Mouse MI	CXCL12, Nrf2	ADSCs	EPC	The exosomes from *SIRT1*-overexpressing ADSCs can restore the function of cell migration and tube formation and recruitment of EPCs to the repair area through Nrf2/CXCL12/CXCR7 signaling.	[84]
Rat MI	miR-21	EnMSCs	HUVEC	EnMSCs showed superior cardioprotection through angiogenic effects via the PTEN/Akt pathway.	[73]
Rat MI	miR-133a-3p	hUC-MSCs	HUVEC	Exosomes from MIF-engineered hUC-MSCs enhanced proliferation, migration, and angiogenesis.	[85]
Rat MI	lncRNA H19	BMSCs	HUVEC	Exosomes from atorvastatin preconditioned MSCs can regulate the expression of miR-675 and activation of VEGF and intercellular adhesion molecule-1 to promote angiogenesis.	[87]
Rat MI	Sfrp2	hUC-MSCs	HUVEC	TIMP2-modified hUC-MSC-derived exosomes can promote HUVEC proliferation, migration, and tube formation in vitro and angiogenesis in rat MI model.	[86]
Rat MI	PDGF-D	hUC-MSCs	HUVEC	Exosomes derived from *Akt*-modified hUC-MSCs resulted in more effective angiogenesis through PDGF-D secretion.	[88]
Limited inflammation					
Mouse I/R	miRNA-181a	hUCB-MSCs	PBMC	Overexpression of miRNA-181a in hUCB-MSC-derived exosomes suppressed inflammatory response in the PBMCs and promoted Treg cell polarization through targeting c-Fos.	[91]
Mouse I/R	miR-182	BMSCs	Raw264.7	BMSC-derived exosomes mediated macrophage polarization by targeting toll-like receptor 4.	[90]
Mouse MI	LPS-primed exosomes	BMSCs	Raw264.7	Exosomes obtained from LPS preconditioning BMSCs strongly increased M2 macrophage polarization and attenuated the postinfarction inflammation in the MI model through inhibition of LPS-dependent NF-*κ*B signaling pathway and activation of the AKT1/AKT2 signaling pathway.	[92]
Vitro model	miR-10a	AD-MSCs	Naïve T cells	miR-10a-loaded exosomes from AD-MSCs facilitated Th17 and Treg responses while reduced that of Th1 in spleen-derived naïve T cells.	[94]
Vitro model	miR-34a, miR-124, and miR-135b	AD-MSCs	THP-1	Melatonin-stimulated exosomes derived from AD-MSCs promoted M2 macrophage differentiation and exerted superior anti-inflammatory response.	[93]
Vitro model	IDO	hUC-MSCs	PBMC	Exosomes from TGF-*β* and IFN-*γ*-stimulated hUC-MSCs significantly promoted the transformation of mononuclear cells to Tregs through IDO regulation.	[95]
Cardiac remodeling					
Rat MI	miR-29 and miR-24	BMSCs	Fibroblast BJ cells	BMSC-secreted exosomes enhanced cardiac repair by transferring miR-29 and miR-24 to fibroblasts.	[17]
Rat MI	Sfrp2	hUC-MSCs	Fibroblast	TIMP2-modified hUC-MSC-derived exosomes decreased TGF-*β*-induced MMP2, MMP9, and *α*-SMA secretion in cardiac fibroblasts and inhibit ECM remodeling.	[86]
Vitro model	miR-21, miR-23a, miR-125b, and miR-145	hUC-MSCs	Fibroblast	hUC-MSC-derived exosomes suppressed myofibroblast formation by inhibiting excess *α*-smooth muscle actin and collagen deposition via the activity of the TGF-*β*/SMAD2 signaling pathway.	[102]

AD-MSCs: adipose mesenchymal stem cells; Mecp2: methyl CpG binding protein 2; HUVEC: human umbilical vein endothelial cells; VEGF: vascular endothelial growth factor; EGF: epidermal growth factor; FGF: fibroblast growth factor; VEGF-R2 and VEGF-R3: receptors of vascular endothelial growth factor; MCP-2: monocyte chemoattractant protein 2; MCP-4: monocyte chemoattractant protein 4; PBMC: peripheral blood mononuclear cells; hUC-MSCs: human umbilical cord-derived mesenchymal stem cells; BMSCs: bone marrow mesenchymal stem cells; CTGF: connective tissue growth factor; NRCM: neonatal rat cardiac myocytes; MIF: macrophage migration inhibitory factor; EnMSCs: human endometrium-derived mesenchymal stem cells; PTEN: phosphatase and tensin homolog; ADRC: adipose-derived regenerative cells; CM: cardiomyocytes; I/R: ischemia-reperfusion; ADSCs: adipose-derived stem cells; HcBMSCs: hypoxia-conditioned bone marrow mesenchymal stem cells; AMI: acute myocardial infarction; ADSC-CM: adipose-derived stem cell conditioned medium; NMCM: neonatal mouse cardiomyocytes; hiPSC: human-induced pluripotent stem cell; EGR1: early growth response factor 1; Mecp2: methyl CpG binding protein 2; lncRNA: long noncoding RNA; hUCB-MSCs: human umbilical cord blood-derived MSCs; EPCs: endothelial progenitor cells; PDGF-D: platelet-derived growth factor D; CXCL12: C-X-C motif chemokine 12; Nrf2: nuclear factor E2 related factor 2; Sfrp2: secreted frizzled- (Fz-) related protein 2; TIMP2: tissue matrix metalloproteinase inhibitor 2; MMPs: matrix metalloproteinases; ceRNA: competitive endogenous RNA; LPS: lipopolysaccharide.
